# Self-Harm among Young People Detained in the Youth Justice System in Sri Lanka

**DOI:** 10.3390/ijerph15020209

**Published:** 2018-01-26

**Authors:** Lushan V. Hettiarachchi, Stuart A. Kinner, Holly Tibble, Rohan Borschmann

**Affiliations:** 1 Forensic Psychiatric Unit, National Institute of Mental Health, Colombo 10620, Sri Lanka; lushhetti@gmail.com; 2 Centre for Adolescent Health, Murdoch Children’s Research Institute, Melbourne 3052, Australia; s.kinner@unimelb.edu.au; 3 Centre for Mental Health, Melbourne School of Population of Global Health, University of Melbourne, Melbourne 3010, Australia; holly.tibble@unimelb.edu.au; 4 Mater Research Institute-UQ, University of Queensland, Brisbane 4072, Australia; 5 Griffith Criminology Institute, Griffith University, Brisbane 4111, Australia; 6 School of Public Health and Preventive Medicine, Monash University, Melbourne 3800, Australia; 7 Health Service and Population Research Department, Institute of Psychiatry, Psychology & Neuroscience, King’s College London, London SE58AF, UK; 8 Department of Psychiatry, University of Melbourne, Melbourne 3010, Australia

**Keywords:** self-injurious behaviour, youth justice, Sri Lanka, adolescence, detention

## Abstract

Self-harm is prevalent in incarcerated adults, yet comparatively few studies of self-harm in detained youth (and even fewer in low- and middle-income countries) have been published. We examined the prevalence and correlates of self-harm in a sample of 181 young people (mean age 15.0 years, SD = 2.3) detained in the youth justice system in Sri Lanka. Structured face-to-face questionnaires assessed demographic characteristics, family and social background, substance use, self-harm history (including frequency, method, and intention), bullying victimization, physical and sexual abuse (victimization and perpetration), and exposure to self-harm/suicide by others. Seventy-seven participants (43%) reported a lifetime history of self-harm, 19 of whom (25%) who reported doing so with suicidal intent. Fifty participants (65% of those with a history of self-harm) reported engaging in self-harm impulsively, with no prior planning. A history of self-harm was associated with being female, prior sexual abuse victimization, prior exposure to self-harm by friends, and a lifetime history of self-harm ideation. High rates of substance use, bullying victimization, parental incarceration, and exposure to suicide were reported across the sample. Young people detained in the youth justice system in Sri Lanka are a vulnerable group with high rates of self-harm, substance use, and psychosocial risk factors. Strategies for identifying and preventing self-harm, and targeted psychological interventions designed specifically to address impulsivity, may contribute to more positive outcomes in this marginalised population.

## 1. Introduction

Self-harm and suicidal behaviours are common in adolescence in both high-income countries [1,2,3,4,5] and low- and middle-income countries [6,7,8] including Sri Lanka [9,10]. Risk factors for self-harm include a history of adversity in childhood, mental illness, and substance use [11], all of which are disproportionately prevalent in young people who come into contact with the youth justice system [12,13]. Most previous research has been conducted in high income countries, and has documented that young people who cycle through the youth justice system often have life trajectories characterized by multiple disadvantage, instability, parental incarceration, abuse and neglect [14,15,16,17,18]. Many have grown up in circumstances of socio-economic deprivation or have been placed in out-of-home care [19,20,21,22]. They are a vulnerable group who, when compared to their peers with no justice contact, have elevated rates of self-harm [23,24,25,26,27], mental disorders [13,28], substance use [12,29,30], poor physical and oral health, and myriad social risk factors including academic disengagement, violent victimization, sexually transmitted infections, and HIV/AIDS risk-taking behaviours [3,12,16,23,31,32]. As such, justice-involved young peopleare a vulnerable group with unique and significant psychosocial needs.

While there has been some research into self-harm in Sri Lanka [33,34,35,36,37,38,39,40,41,42,43,44], it has largely focused on adults, and there are no published accounts of the epidemiology of self-harm in young people in detention in the youth justice system in Sri Lanka. Societal and cultural differences between low- and middle-income countries- such as Sri Lanka- and high-income countries could have a significant impact on the epidemiology of self-harm in young people [6], particularly in vulnerable groups such as those involved in the youth justice system. This missing knowledge is a prerequisite for informing targeted, evidence-based responses to reduce the risk of self-harm in this marginalized and vulnerable population. The primary aim of this study was to estimate the prevalence of self-harm in young people in detention in the youth justice system in Sri Lanka. The secondary aim was to examine the risk and protective factors associated with self-harm.

## 2. Materials and Methods

### 2.1. Setting and Participants

In the youth justice system in Sri Lanka, one option available to judges when sentencing young people (aged 12–16) convicted of any offence is to recommend rehabilitation at a certified school for a set period of three years (although many young people remain in such schools for considerably longer than this period). There are four fully functioning certified schools in Sri Lanka: two in the Western province (Makola and Ranmuthugala), one in the Southern province (Hikkaduwa), and one in the Central province (Keppetipola). All certified schools are administered by the Department of Probation and Child Care Services under the Ministry of Social Services [45]. Young people detained in certified schools continue their formal education during this time, with many undertaking various forms of vocational training such as welding, woodwork, or sewing.

Certified schools also house young people in need of special care with no justice system involvement, such as those in need of protective custody, and victims of abuse, trafficking or other exploitation [45]; these young people were excluded from our study. Additionally, potential participants were excluded from the study if:

1. They had a diagnosed intellectual disability that prevented them from understanding fully the questions asked of them;
2. Their parent or guardian did not provide informed consent allowing them to participate;
  All eligible young people in these four certified schools were invited to participate in the study.

### 2.2. Data Collection

#### Study Instrument and Measures

All data were collected by the lead author (LVH), an independent psychiatrist with experience working in youth justice settings. Data were collected during structured, face-to-face interviews exploringeach participant’s demographic characteristics, family and social background, substance use history, self-harm history, bullying victimization, and physical and sexual abuse (victimization and perpetration). Demographic variables included age, sex, and ethnicity (self-reported as Sinhalese, Tamil or Muslim). Family and social background included parents’ marital status, parents’ substance use, family history of suicide, and family history of incarceration. Substance use history included the types of substances used, age at first use, first substance ever used, and frequency of use prior to detention. Self-harm history included any prior self-harm, age at first self-harm, behavioural intent of self-harm, the method(s) of self-harm used, the frequency of self-harm, the expected outcome, and whether the participant knew anyone who had died by suicide. Bullying victimisation included assessing for physical, emotional, and verbal bullying, and the location of previous bullying. Physical and sexual abuse items included assessing for any prior physical or sexual abuse, age at which this commenced, number of perpetrators and whether they were known to the participant, and whether the participant had self-harmed because of this abuse. The questionnaire was piloted extensively to ensure that all questions were easy to comprehend and unambiguous.

### 2.3. Outcome Measures

The primary outcome was a history of any self-harm, as reported by participants. The definition of self-harm used in this study was adapted from Madge and colleagues [46]: “an act with a non-fatal outcome in which an individual deliberately initiates behaviour (such as self-cutting), or ingests an illicit drug or non-ingestible substance or object, with the intention of causing harm to themselves”’ (p. 669). We also included poisoning with any legal substance, in accordance with our previous large-scale epidemiological study of self-harm [1,47,48]. Secondary outcome measures included contextual factors related to previous self-harm, including the method(s) of self-harm used, the behavioural intent associated with self-harm, the reasons for self-harm, and the number of lifetime self-harm episodes. We used Bridge and colleagues’ [49] definition of suicidal ideation: “Passive thoughts about wanting to be dead or active thoughts about killing oneself, not accompanied by preparatory behavior.”.

### 2.4. Procedure

Informed written consent was obtained from a parent or guardian of the young person prior to entering the study. The questionnaire was administered in all four certified schools in Sri Lanka. Interviews were conducted in private interview rooms and data were anonymized at the point of data entry. All study data were collected between 1 July 2016 and 30 September 2016. Ethical clearance was granted from the Ethics Review Committee of the Faculty of Medicine at the University of Ruhuna, Sri Lanka (reference no: 31/05/2016:3.20).

### 2.5. Data Analysis

Descriptive statistics were used to examine participant characteristics and the prevalence and characteristics of self-harm. When comparing participants’ self-reported self-harm and sex, Fisher’s exact test was used for single-response categorical variables (such as sex), and proportion tests were used for multiple-choice categorical variables (such as bullying type). For variables which were found to be non-independent of self-harm incidence, logistic regression was used to assess the effect size. All analyses were conducted using Stata v14.2 (StataCorp LLC, College Station, TX, USA) [50].

## 3. Results

### 3.1. Profile of Cohort Members

One hundred and eighty-one young people (92.3% of the 196 eligible residents at the four certified schools) provided informed consent and participated in the study. Their demographic characteristics are shown in Table 1. Most participants were male, Sinhalese, and aged 16 or above (reflecting the fact that many people remain in detention for considerably longer than the recommended three months). Seventy-seven (43%) reported a lifetime history of self-harm. More than half of all participants had substance use histories and two thirds reported being the victim of verbal bullying. One-in-six reported childhood sexual abuse victimization, more than one quarter reported a family history of suicide, and more than half of participants (107; 59%) reported experiencing self-harm ideation at some point in their lives. The offences that cohort members were convicted of included theft (38%), truancy from school (19%), eloping (13%), selling drugs (8%) and sexual offences (4%). Missing data was minimal: 2.8% for sexual abuse history, 1.1% for ethnicity, and 0.6% for substance use history. All other variables had complete data (non-calculable for multiple choice responses such as bullying type).

### 3.2. Factors Associated with Self-Harm

Participants who had self-harmed were more likely to be female (OR = 3.58, 95% CI = 1.82–7.07), to have been the victim of sexual abuse (OR = 2.89, 95% CI = 1.33–6.24), to have been exposed to self-harm by one or more friends (OR = 2.19, 95% CI = 1.06–4.53), or to be detained in the Ranmuthugala certified school (OR = 3.85 compared to Hikkaduwa, 95% CI = 1.76–8.43). Table 2 identifies contextual factors relating to self-harm in the 77 participants who reported a lifetime history of self-harm.

The most common method of self-harm reported was cutting (84%) and the mean number of reported lifetime self-harm episodes was 10.1 (SD: 16.0; range 1–100; median: 5). More than one third of participants who had self-harmed reported doing so for the first time since being detained in their certified school. In relation to their most recent self-harm episode, one quarter (19/77; 25%) of participants stated that they believed they would die and almost two thirds (50/77; 65%) reported engaging in self-harm impulsively, without any prior planning. Of the 77 participants who reported self-harm, females were more likely than males to report cutting (*p* = 0.046), poisoning (*p* = 0.004), and to report that the expected outcome from their most recent self-harm episode was death (*p* = 0.001). Males were more likely than females to report banging their head (*p* = 0.016). The most commonly reported reasons for self-harm included anger, sadness, family problems, and frustration. Other reasons—including to threaten others, to solve problems, in response to scolding, experimenting, and self-harming due to intoxication from illicit substances—were reported less frequently.

## 4. Discussion

In our study, more than two-in-five (43%) young people detained in certified schools in Sri Lanka had engaged in self-harm at some point in their lives and, of these, more than one third did so for the first time since being detained in a certified school. A lifetime history of self-harm was associated with being female, prior sexual abuse victimization, prior exposure to self-harm by friends, and a lifetime history of self-harm ideation. Young people detained in the Ranmuthugala certified school were more likely than their peers in other schools to have self-harmed, likely reflecting that Ranmuthugala was the only school of the four which detained females at the time of data collection. Of the young people with a history of self-harm, two thirds reported cutting themselves as the method of self-harm and one quarter (10% of the *entire* sample) reported self-harming with suicidal intent in relation to their most recent episode. This figure was twice as high as that reported in a youth justice sample in Australia [23], and orders of magnitude higher than that observed in community samples, in which the lifetime prevalence of self-harm with suicidal intent is approximately 1% [1]. The prevalence of self-harm was almost twice as high in females as in males (64% vs. 33%), a finding which was consistent with previous epidemiological studies of self-harm both in the general population [1] and in youth justice samples [23]. This finding may also possibly reflect the fact that females in this setting represent a particularly vulnerable subset of females from the wider community.

More than half of our participants (59%) reported experiencing self-harm ideation at some point in their lives.

Almost two thirds of participants with a history of self-harm in our sample reported having self-harmed prior to their current detention in acertified school. This finding is similar to that from studies conducted in high-income countries [23] and highlights the importance of screening young people for a history of self-harm upon reception into detention. Previous research has demonstrated that the risk of suicide deaths in juvenile detention is the lowest when young people are screened for a history of self-harm and current suicidal ideation within 24 h of reception into detention [51]. Our finding that a history of self-harm was associated with prior exposure to self-harm by friends underscores the importance of engaging peers and exploring peer-supportoptions in interventions designed to reduce self-harm. The high degree of impulsive self-harm reported by participants in our sample (65% reported no planning at all and 85% reported less than three hours’ planning) suggests that systematic preventive interventions may have more impact than targeted, clinical responses to emergent risk.

Participants in our study reported a high prevalence of sexual abuse, bullying victimization, and a range of parental risk factors including incarceration, substance use history, parental self-harm, family conflict, and parental abuse, highlighting the complex adversity that characterizes many young people in detention [52]. Many of these have previously been identified as adverse childhood experiences (ACEs) which are associated with an increased risk of self-harm and suicide in psychiatric populations [53,54] and the general population of other LMICs [55]. ACEs are more prevalent in justice-involved young people than in the general population [56], and are associated with both self-harm and violence perpetration in the general population [57]. As such, in addition to proximal prevention efforts, there is a need for upstream efforts to reduce exposure to adversity and increase resilience, to reduce the impact of self-harm in this vulnerable population of young people. One avoidable consequence of employing a narrow conceptualization of criminal offending in this population is that it fails to contextualize what is often a lifetime of disadvantage, trauma and neglect [14,15,16,17,18].

Given that self-harm in young people may be a conspicuous marker of emotional and behavioural problems that are often associated with adverse life outcomes [48,58,59], it seems that the increased risks associated with self-harm do not attenuate upon discharge from the youth justice system. Findings from previous studies have indicated that the period of greatest risk for this population is likely to be after release from detention [60,61,62]. As such, research is urgently needed to explore strategies for ongoing support and prevention of self-harm and other adverse health outcomes, perhaps in conjunction with investments made by the criminal justice system to reduce the risk of future offending. Research examining the health and psychosocial trajectories of young people in and released from detention in low- and middle-income countries is also needed, and should be considered a research priority. Bearing in mind the strong association between self-harm and reduced life expectancy [58,63,64], one feasible avenue for obtaining such data may be studies of mortality in young people following discharge from youth justice detention in low- and middle-income countries.

Our findings need to be considered in the context of some potential limitations. First, our data were cross-sectional and as such we cannot infer causality. Second, self-harm data were obtained during face-to-face interviews and may have been influenced by reporting biases such as social desirability bias [65]. Our estimates of self-harm-in terms of both the prevalence and the strength of associations with other variables—are likely to be conservative, as most self-harm occurs in private and is never reported [66]. Finally, we did not use a standardised tool to assess mental disorders, relying instead on a brief clinical interview. It is likely that this method resulted in considerable under-ascertainment of mental disorder in our sample.

Our study also contained several notable strengths. First, to our knowledge, ours is the first published estimate of the prevalence of self-harm in young people detained in the youth justice system in Sri Lanka. Data relating to adolescent self-harm in low- and middles-income countries are scarce 66 [6,67], but data from adolescents detained in the justice system in such countriesis non-existent in the published literature, highlighting the novel nature of our findings. Second, our response rate of 92% was very high and our sample was representative of all young people convicted of similar offences during the data collection period. Third, we assessed a large number of variables to identify factors associated with self-harm in young people detained in certified schools under the youth justice system, an approach mirroring previous research involving justice-involved young people in high-income countries [23,26].

## 5. Conclusions

Our study identified a high prevalence of self-harm, self-harm ideation, and self-harm with suicidal intent, among young people detained in the youth justice system in Sri Lanka. The prevalence of these behaviours was higher than that reported in youth justice samples from high-income countries [16,23,26], which are, in turn, markedly higher than in general population studies [1,61]. Young people who were female, who had been the victim of sexual abuse, and who had been exposed to self-harm by friends were more likely to report self-harm [48,58,59,60,61,62]. Targeted research is urgently needed to minimise self-harm and its consequences in this population, both during and after their detention in the youth justice system [58,63,64].

## Figures and Tables

**Table 1:** Sample characteristics according to self-harm history in 181 justice-involved young people detained in certified schools in Sri Lanka.

Characteristic/Risk Factor	Response	Self-Harm (*n* = 77)	No Self-Harm (*n* = 104)	*p*-Value for Difference between Groups
		*n* (row %)	*n* (row %)	
Sex	Male	44 (34%)	86 (66%)	<0.001 ^3^
	Female	33 (65%)	18 (35%)	
Ethnicity	Sinhalese	69 (45%)	83 (55%)	0.159 ^3^
	Tamil	5 (25%)	15 (75%)	
	Muslim	2 (29%)	5 (71%)	
Age in years	<14	13 (29%)	32 (71%)	0.100 ^3^
	14–15	10 (45%)	12 (55%)	
	>15	54 (47%)	60 (53%)	
School	Hikkaduwa	20 (32%)	42 (68%)	0.001 ^3^
	Keppetipola	10 (29%)	25 (71%)	
	Makola	14 (42%)	19 (58%)	
	Ranmuthugala	33 (65%)	18 (35%)	
Substance use history	Yes	44 (45%)	53 (55%)	0.445 ^3^
	No	33 (40%)	50 (60%)	
Bullying victimization	Yes	55 (42%)	75 (58%)	1.000 ^3^
	No	22 (43%)	29 (57%)	
Bullying type ^1^	Physical	33 (49%)	34 (51%)	0.161 ^4^
	Psychological	47 (49%)	49 (51%)	0.064 ^4^
	Verbal	50 (41%)	71 (59%)	0.638 ^4^
Diagnosed mental illness ^1^	Anxiety	2 (40%)	3 (60%)	0.907 ^4^
	Depression	5 (71%)	2 (29%)	0.115 ^4^
	Schizophrenia	3 (75%)	1 (25%)	0.184 ^4^
Sexual abuse victimization	Yes	21 (62%)	13 (38%)	0.007 ^3^
	No	51 (36%)	91 (64%)	
Parents’ marital status	Married	25 (35%)	46 (65%)	0.257 ^3^
	Separated/divorced	16 (44%)	20 (56%)	
	Don’t know	36 (49%)	38 (51%)	
History of parental incarceration ^1^	Father	19 (51%)	18 (49%)	0.224 ^4^
	Mother	4 (36%)	7 (64%)	0.669 ^4^
Family ^2^ history of suicide	Yes	22 (42%)	30 (58%)	1.000 ^3^
	No	55 (43%)	74 (57%)	
Exposure to self-harm by ≥1 friends	Yes	64 (47%)	72 (53%)	0.037 ^3^
	No	13 (29%)	32 (71%)	
Lifetime history of self-harm ideation	Yes	77 (72%)	30 (28%)	<0.001 ^3^
	No	0 (0%)	74 (100%)	

^1^ Not mutually exclusive; ^2^ First or second degree relative; ^3^ Fisher’s exact test ignoring missing data; ^4^ Test of proportions by response where multiple responses are available.

**Table 2:** Self-harm characteristics by gender reported by 77 young people detained in certified schools in Sri Lanka.

Characteristic	Response	Male (*n* = 44)	Female (*n* = 33)	*p*-Value for Difference between Groups
		*n* (row %)	*n* (row %)	
First self-harm episode prior to certified school?	Yes	31 (66%)	16 (34%)	0.056 ^2^
	No	12 (41%)	17 (59%)	
Method of self-harm used ^1^	Cutting	34 (52%)	31 (48%)	0.046 ^3^
	Poisoning	4 (25%)	12 (75%)	0.004 ^3^
	Banging head	7 (100%)	0 (0%)	0.016 ^3^
	Strangling	1 (33%)	2 (67%)	0.395 ^3^
	Other	0 (0%)	2 (100%)	0.098 ^3^
Expected outcome of most recent self-harm episode	Definitely die	9 (47%)	10 (53%)	0.001 ^2^
	Might die	1 (10%)	9 (90%)	
	Will not die	33 (70%)	14 (30%)	
Time spent planning most recent self-harm episode	No planning	28 (56%)	22 (44%)	0.395 ^2^
	<3 h	10 (71%)	4 (29%)	
	>3 h	6 (46%)	7 (54%)	
Number of lifetime self-harm episode	≤10	40 (65%)	22 (35%)	1.000 ^2^
	11–50	8 (62%)	5 (38%)	
	≤51	1 (50%)	1 (50%)	
Reasons for self-harm ^1^	Anger	14 (58%)	10 (42%)	0.887 ^3^
	Sadness	11 (48%)	12 (52%)	0.281 ^3^
	Family problems	3 (30%)	7 (70%)	0.063 ^3^
	Frustration	2 (33%)	4 (67%)	0.220 ^3^
	Other	17 (49%)	18 (51%)	0.165 ^3^

^1^ Not mutually exclusive; ^2^ Fisher’s exact test ignoring missing data; ^3^ Test of proportions by response where multiple responses are available.
